# Fairness-aware recommendation with meta learning

**DOI:** 10.1038/s41598-024-60808-x

**Published:** 2024-05-02

**Authors:** Hyeji Oh, Chulyun Kim

**Affiliations:** Department of IT Engineering, Sookmyung Women’s University, 100 Cheongpa-ro 47-gil, Yongsan-gu, Seoul, 04310 Korea

**Keywords:** Recommender systems, Fairness, Cold-start recommendation, Meta-learning, Deep learning, Artificial intelligence, Engineering, Mathematics and computing

## Abstract

Fairness has become a critical value online, and the latest studies consider it in many problems. In recommender systems, fairness is important since the visibility of items is controlled by systems. Previous fairness-aware recommender systems assume that sufficient relationship data between users and items are available. However, it is common that new users and items are frequently introduced, and they have no relationship data yet. In this paper, we study recommendation methods to enhance fairness in a cold-start state. Fairness is more significant when the preference of a user or the popularity of an item is unknown. We propose a meta-learning-based cold-start recommendation framework called FaRM to alleviate the unfairness of recommendations. The proposed framework consists of three steps. We first propose a fairness-aware meta-path generation method to eliminate bias in sensitive attributes. In addition, we construct fairness-aware user representations through the meta-path aggregation approach. Then, we propose a novel fairness objective function and introduce a joint learning method to minimize the trade-off between relevancy and fairness. In extensive experiments with various cold-start scenarios, it is shown that FaRM is significantly superior in fairness performance while preserving relevance accuracy over previous work.

## Introduction

Recommender systems^1^ have become necessary in e-commerce, social web, and subscription platforms to retain existing users or attract new users. In traditional collaborative filtering techniques^2–6^, sparse interaction matrices for new users or items make cold-start recommendations difficult. Previous works^7–10^ utilize demographic information such as gender, age, and occupation to alleviate the cold-start problem. These studies assume that users with the same sensitive attributes (e.g., gender) may have similar behavioral patterns. However, this assumption has limitations that lead to unfair recommendations^11^.

**Figure 1:**
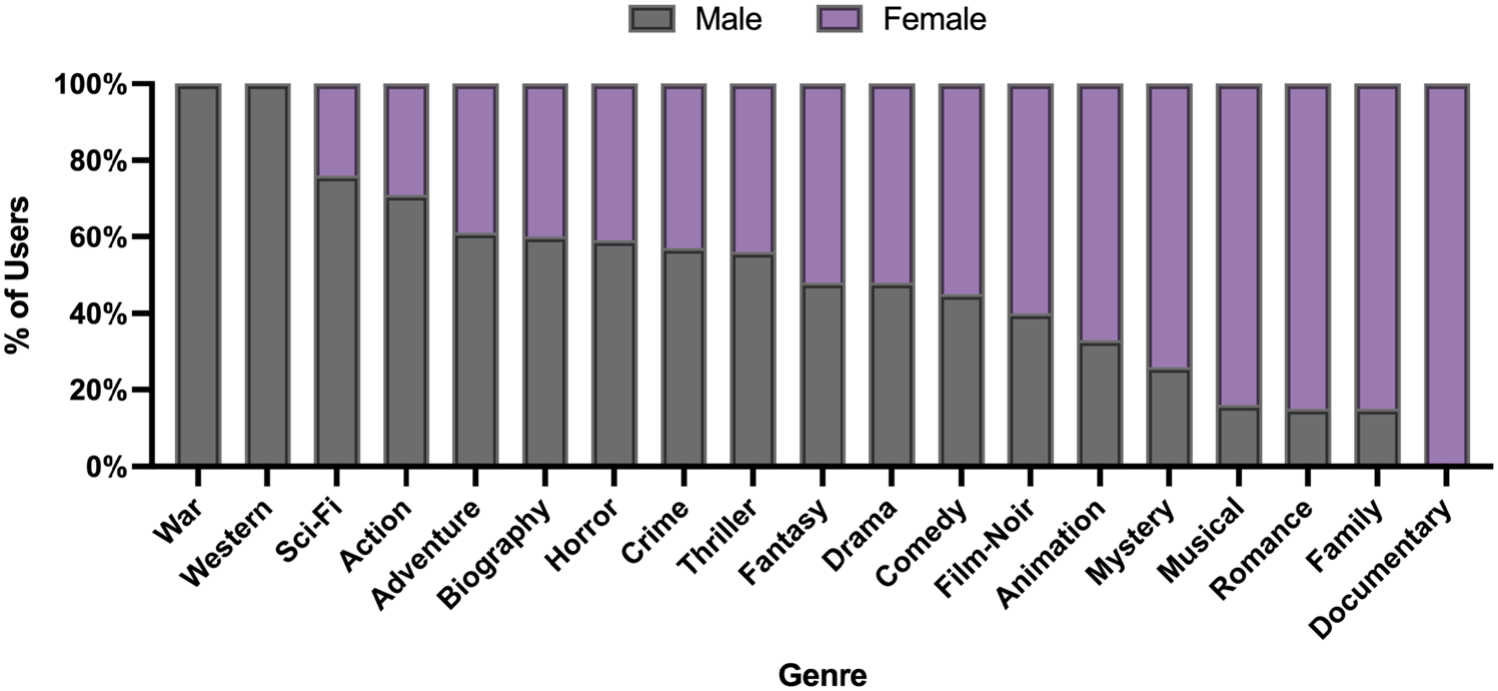
Gender distribution by genre of each user’s preference for the *Movielens 1M* dataset. We define each user’s preferred genre as a genre with a proportion of more than 10% of the movies with which users interacted.

In Fig. 1, it is shown the unfair distribution of genre preference by gender in Movielens 1M^12^. We count only genres that account for more than 10% of the movies rated by each user. Surprisingly, none of the female users in the dataset favored the *War* and *Western* genres. The preference ratio for *Action* and *Sci-Fi* movies is more than 70% for male users, and *Adventure*, *Crime*, *Horror*, and *Thriller* films also appear to attract more attention from male users than female users. On the other hand, none of the male users enjoyed *Documentary* movies. The *Romance* and *Family* genres also show a strong gender bias, as female users’ preferences account for more than 80%. Similarly, *Animation*, *Musical*, and *Mystery* movies have much higher preferences for female users than male users, as shown in Fig. 1. Therefore, the cold-start recommendation models learned from the user’s profile data (e.g., gender) have limitations in recommending War or *Action* movies rather than *Romance* to male users who want *Romance*. In other words, some biases in training data induce unfairness problems^13,14^ in recommender systems.

**Figure 2:**
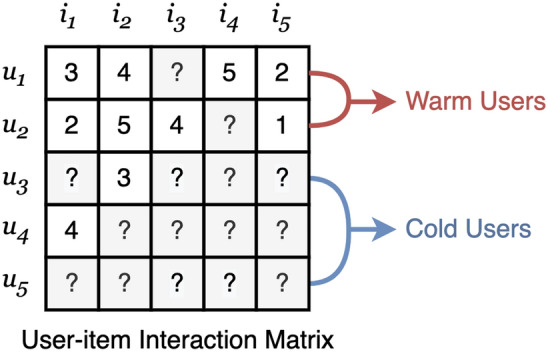
User-item interaction matrix with warm users and cold users. $u_1$ and $u_2$ are warm users with two or more interacted items. $u_3$, $u_4$, and $u_5$ are cold users with one or less interacted items.

**Figure 3:**
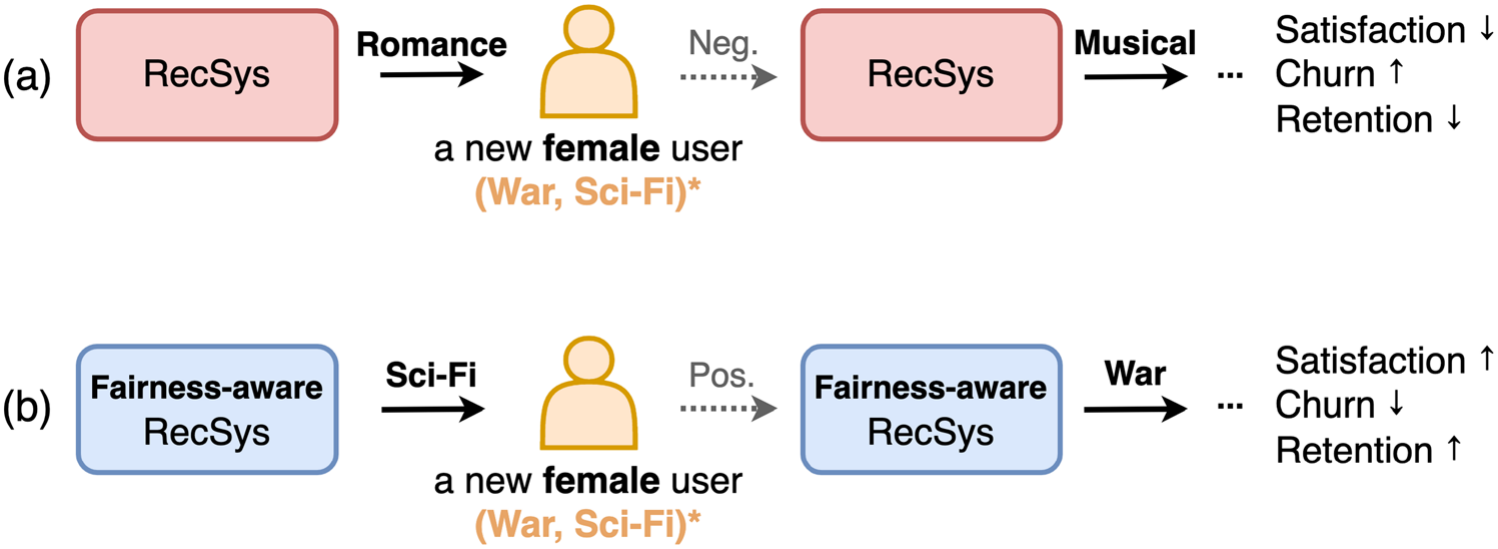
(**a**) Cold-start recommender system without removing the gender bias. (**b**) Fairness-aware cold-start recommender system.

Recently, several existing works^11,15–18^ have been paying attention to enhancing fairness. The previous study of fairness in recommender systems has only assumed non-sparse user-item interaction with existing users and items, i.e., warm-start state. However, this paper captures that improving fairness in a cold-start is more critical than in a warm-start. If user-item interaction data is sufficient, the recommendation model can learn the personalized characteristics of each user. For example, in Fig. 2, user $u_1$, $u_2$ are existing users (i.e., warm-start users) with non-sparse user-item interaction data, and u3, u4, and u5 are new users (i.e., cold-start users) with sparse user-item interaction data. we suppose that the recommendation model predicts user $u_2$’s preference for item $i_4$. The recommendation model can predict that user $u_1$ will have a similar preference to user $u_2$ for item $i_4$ because they rated items $i_1$, $i_2$, and $i_5$ similarly. In this way, in a warm-start state (i.e., non-sparse user-item interaction), it is easy to capture the personalized preference of each user. However, sparse user-item interaction data is challenging to learn user preferences due to the lack of historical feedback data. Previous research^19^ solves this cold start problem by recommending popular items or recommending items preferred by users with the same user characteristics (gender, age, occupation, etc.). However, it is essential to mitigate data bias in these attributes, such as gender bias, as shown in Fig. 1. This is why enhancing fairness in cold-start is more important than improving fairness in warm-start. Zhu et al.^20^ captured the importance of unbiased recommendations for new items and proposed a learnable framework that eliminates popularity bias in the item cold-start scenario. However, the framework might have a limitation only considering the popularity bias of individual items and ignoring bias in sensitive attributes such as gender. This limitation leads to some problems of recommending unwanted items to new users only with demographic information, not feedback data. As shown in Fig. 3a, the recommendation algorithm, unaware of fairness, recommends *Romance* movies to new female users who prefer *War* and *Sci-Fi* without removing the gender bias. The problem is that it takes a considerable amount of time for the system to learn the flavor of the new user, which may eventually lead to the churn of the new user. In contrast, the fairness-aware recommender system in Fig. 3b without gender bias improves user satisfaction by recommending *Sci-Fi* and *War* films that the user likes as soon as they use the platform. Therefore, fairness for sensitive attributes in the cold-start state is vital in keeping new users and increasing heavy users.

This paper proposes a novel framework called FaRM (Fairness-aware Recommendation with Meta-learning), which reduces bias for sensitive attributes of users or items and can also adapt to cold-start states. Previous works related to meta-learning-based recommendations^9,10^ have alleviated the cold-start problem, but the unfairness problem remains unresolved. Our study aims to enhance the fairness of the meta-learning-based recommendation framework to overcome the limitations of previous works.

### Contributions

The key contributions of our work can be summarized as:

- It is the first attempt to improve the fairness of the cold start recommendation model, which recommends items to new users reasonably.
- We propose a novel fairness-aware framework named FaRM, which enhances fairness in a meta-learning-based model. We introduce a novel meta-path generation method that improves fairness through the fairness-aware random walker. We also investigate joint training techniques for minimizing the trade-off between relevance and fairness.
- Extensive experiments demonstrate that FaRM enhances fairness in cold-start scenarios and significantly outperforms various state-of-the-art methods.

The remainder of this paper has the following structure. We discuss existing works on meta-learning and fairness related to FaRM in section “Related work” and formalize the problem of FaRM in section “Problem definition”. In section “Methodology”, we present the proposed recommendation framework, FaRM, and introduce new methods that have introduced fairness to the meta-learning-based cold-start recommender systems. In section “Experiments”, we experimentally evaluate the proposed model. Finally, We conclude our findings and discuss future research in section “Conclusion”.

## Related work

### Cold-start recommendation

Sparse user-item interaction for new users and items (i.e., cold-start recommendation) is one of the challenging problems in collaborative filtering^2,5,21^. Early research focused on content-based filtering^22^, which uses metadata from users and items to solve the cold-start problem. Shi et al.^23^ alleviated the cold-start problem by introducing heterogeneous information networks (HINs)^24^ that embed multiple meta-paths to improve the quality of contents.

The success of meta-learning^25^, which can learn with even a small amount of data, has contributed significantly to solving the cold start problem. Vartak et al.^26^ solved the item cold-start problem by introducing metric-based few-shot learning on recommendation tasks to adapt to new items. Lee et al.^9^ proposed a recommendation framework that improves performance in various cold-start scenarios by applying an optimization-based approach, MAML^27^. Moreover, Lu et al.^10^ proposed a method to solve cold-start problems at both data-level and model-level by applying HIN^23,24^ to the MAML framework. Despite several investigations that reducing bias in cold-start is essential^19,28^, these methods did not consider fairness or de-baising. Therefore, we aim to improve the quality of recommendations by reducing bias for sensitive attributes and improving fairness in the MAML framework^9,10,27^.

### Fair meta-learning

Fairness has become an indispensable problem in machine learning in recent years^13,29^. A small amount of research has recently begun to improve fairness in meta-learning approaches^30–32^. Slack et al.^31^ proposed a fairness-aware online meta-learning framework by adding fairness constraints based on decision boundary covariance (DBC)^33^. Similarly, Zhao et al.^32^ applied fairness-aware constraints to the few-shot image classification task. In addition, Slack et al.^30^ proposed two kinds of fairness regularizers and improved the fairness of the MAML framework^27^ by joint training^34^ between the accuracy loss and the fairness regularizer. However, these approaches focused only on general classification tasks, not recommendation tasks. This paper proposes a novel fairness regularizer suitable for the rating prediction task to reduce bias between different item groups in MAML-based recommender systems^9,10^.

### Fairness-aware recommendation

Fairness has begun to be studied in recommender systems because unfair recommendations can cause fatal damage to users or platforms^11,16,18,20,35^. The fairness in recommendation tasks can be categorized as user-side (i.e., consumer-side) and item-side (i.e., provider-side) fairness^14,36^. In the item-side study, Abdollahpouri et al.^37^ analyzed the impact of popularity bias on different individuals or groups of users. Furthermore, Biega et al.^38^ formalized equity-of-attention fairness that captures the difference between the deserved and received attention in post-processing. Meanwhile, Yao et al.^39^ provided four fairness metrics for group-level fairness on the user-side. Li et al.^40^ provided a fairness constructed re-ranking method to enhance the fairness of different user groups. Islam et al.^15^ proposed a novel fair recommendation network by applying two de-biasing methods for user embeddings to neural collaborative filtering (NCF)^21^. In addition, fairness works have also been proposed from various perspectives, such as multi-side fairness^41,42^, adversarial learning^11,18^, HIN representation learning^17^, re-ranking^43–45^, and in-processing methods^46,47^.

Unfortunately, these methods address fairness in warm-start with existing users and items rather than cold-start. Zhu et al.^20^ captured this limitation of existing works and proposed a learnable re-ranking framework that strengthens fairness in cold-start. However, this framework desires to reduce only the item popularity bias while overlooking the bias for the user’s sensitive attributes. To overcome these limitations of previous works, we aim to de-bias the sensitive attributes by improving the fairness of user-oriented meta-learning tasks^9,10^.

## Problem definition

In this section, we introduce the problem definition of FaRM. This paper is inspired by the HIN-based recommendation models and the definition of HIN is as follows^10,23,24^.

### Definition 1

Heterogeneous information network. We suppose that our dataset is a heterogeneous information network $G=(V, E)$ where *V* denotes the set of nodes and *E* denotes the set of links. A network is associated with a node type mapping function $\phi : V \rightarrow A$ and a link type mapping function $\varphi : E \rightarrow R$, where *A* denotes the set of node types and *R* denotes the set of link types, where $|A| + |R| > 2$.

We propose a novel algorithm to generate a fair meta-path in section “Methodology”, and meta-path is defined as follows^10^.

### Definition 2

Meta-path. We define a meta-path $\textbf{p}$, which generates node sequences, as a path in the form of $\textbf{p} = a_{1} \xrightarrow {r_{1}} a_{2} \xrightarrow {r_{2}} \cdots \xrightarrow {r_{l}} a_{l+1}$, where *l* denotes the length of $\textbf{p}$, each $a_i \in A$ and $r_i \in R$.

We define sensitive attributes for users or items such as gender as follows.

### Definition 3

Sensitive attributes. A sensitive attribute mapping function $\Phi$ is defined as $\Phi : (V, A) \rightarrow S$, where *S* denotes the set of sensitive attributes.

## Methodology

In this section, we introduce a novel fairness-aware recommendation framework, FaRM. Furthermore, we propose various fairness-aware methods of FaRM.

### Overall framework of FaRM

**Figure 4:**
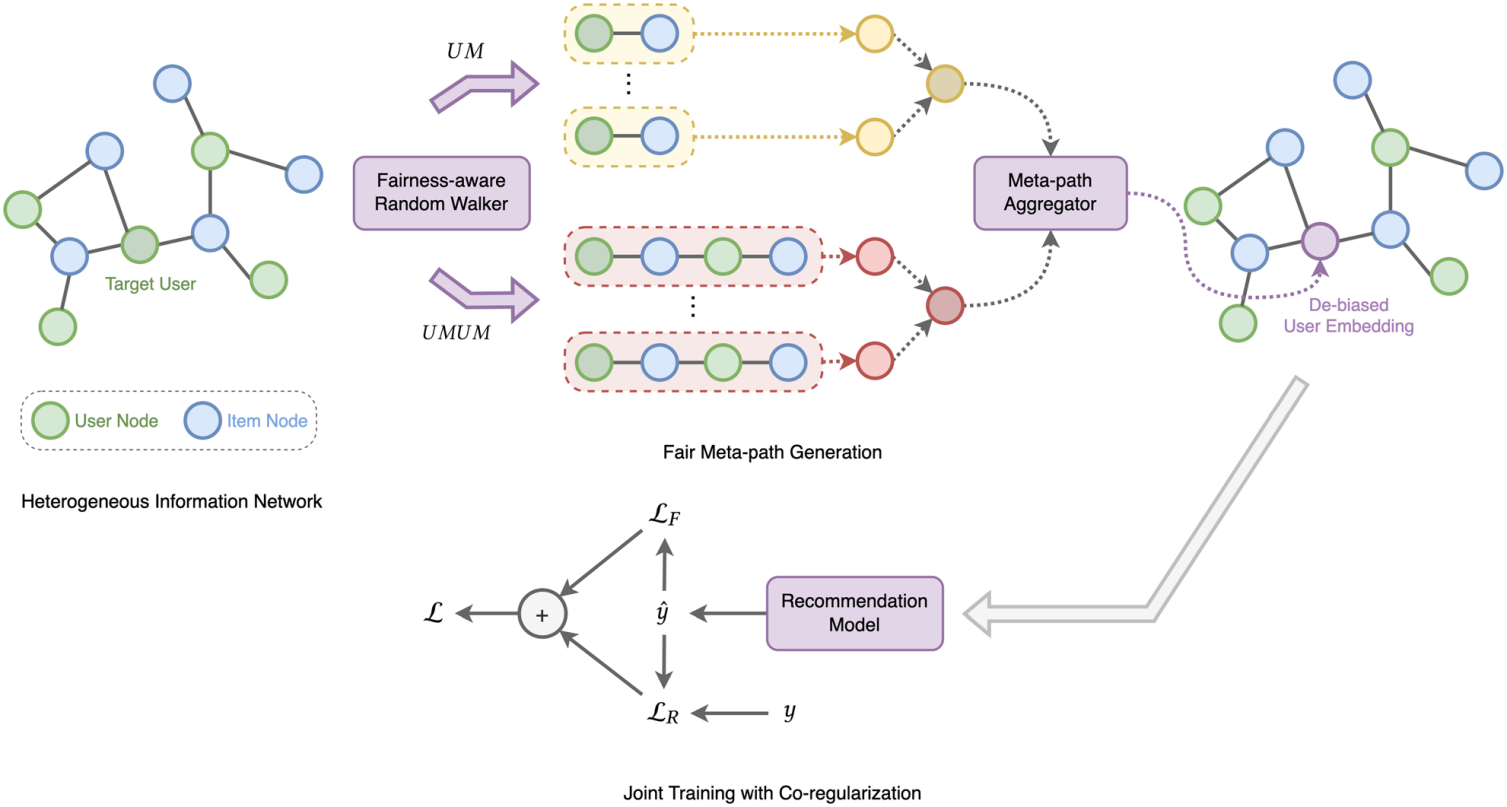
Overall framework of FaRM.

In Fig. 4, it is shown the overall structure of the MAML-based fairness-aware recommendation framework, FaRM, proposed in this paper. Given the set of users $U=\{u_1, u_2, \ldots u_N\}$ and the set of items $I=\{i_1, i_2, \ldots i_M\}$, the task for each user *u* is defined as $\mathcal {T}_u=(\mathcal {S}_u, \mathcal {Q}_u)$, where $\mathcal {S}_u$ denotes the support set of user *u* and $\mathcal {Q}_u$ denotes the query set of user *u*. For each task, the procedure shown in Fig. 4 is performed. First, a fairness-aware random walker creates de-baised meta-paths for a target user *u*. Second, we convert each of the meta-paths into a dense representation. Then, the meta-path aggregator aggregates two types of meta-paths to create a dense user representation $\textbf{x}_u$ that enters the recommendation model *f* as input. Finally, the proposed framework learns the model through joint training^34^ for fairness and relevance objectives.

### Fairness-aware random walker

Several existing works have employed a random walk^48^ to construct meta-paths^49,50^. However, We propose a transition probability for a fair random walk that fairly generates the next node because the random walk cannot capture the bias of the sensitive attributes.

**Figure 5:**
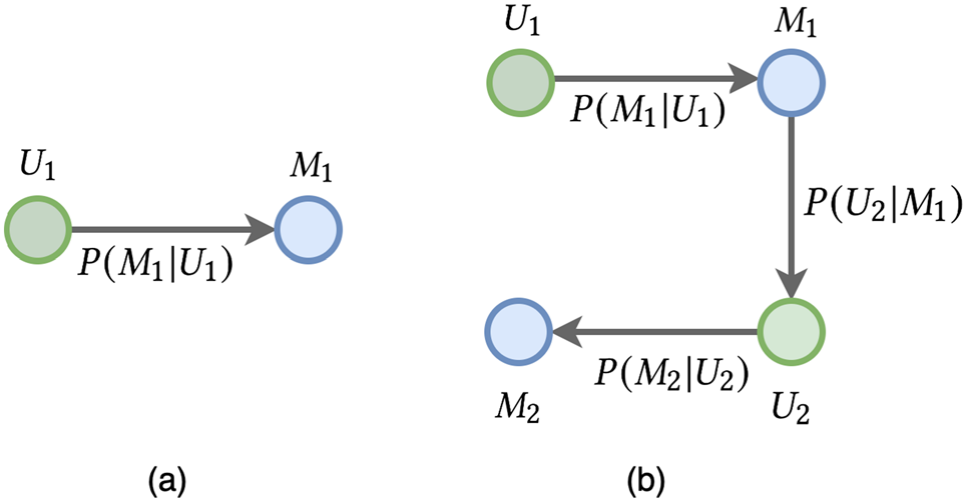
The type of Meta-paths of FaRM. (**a**) An example of meta-path *UM*. (**b**) An example of meta-path *UMUM*.

As shown in Fig. 5, the proposed algorithm generates two types of meta-paths. The type of meta-path $\textbf{P}$ consists of $UM(User-Movie)$ and $UMUM(User-Movie-User-Movie)$, where *UM* encodes the context of “movies rated by the user”, and *UMUM* means the context of “movies rated by another user who has seen the same movie”. The transition probability of a random walker is defined as follows,

1 $$\begin{aligned} P(v_{i+1}|v_{i}) = 1 - P(\Phi (v_{i+1}, a_{i+1})|\Phi (v_{i}, a_{i})) \end{aligned},$$

where $v_{i+1}$ is a neighbor node of $v_i$, $a_i$ and $a_{i+1}$ are sensitive attributes of $v_i$ and $v_{i+1}$ respectively, and $a_{i} \ne a_{i+1}$. For the convenience of explanation, it is assume that *A* in Definition 2 is $A = \{User(U), Movie(M)\}$, where $a_i, a_{i+1} \in A$. In Addition, the sensitive attribute mapping function $\Phi$ in Definition 3 returns a gender value (male or female) if the node type is *User*(*U*) and returns genre values (*Romance*, *Action*, *Thriller*, etc.) if the node type is *Item*(*I*) For example, If $a_{i}$ is the node type *User*, $\Phi (v_{i}, a_{i})$ can be the type of gender, such as male or female. Similarly, if $a_{i}$ is the node type *Movie*, $\Phi (v_{i}, a_{i})$ can be the type of genre, such as *Romance* or *Action*. Equation (1) allows us to select more nodes for disadvantaged groups and fewer nodes for advantaged groups.

#### Example 1

Suppose we are considering a meta path from user A, who is male, to a romance genre movie $\alpha$. Here, $\Phi (v_i, a_i)$ represents the sensitive attribute value of user A, which is ‘male’, and $\Phi (v_{i+1}, a_{i+1})$ represents the sensitive attribute value of movie $\alpha$, which is ‘romance’. The value of $P(\Phi (v_{i+1}, a{i+1})|\Phi (v_i , a_i))$ can be calculated using statistics from the dataset. For example, if this statistical value is calculated from the Movielens 1M dataset^12^, the probability comes out to be 0.1598, and based on this, the transition probability value is calculated as $1 - 0.1598 = 0.8402$. The entire transition probability matrix calculated in this manner from the Movielens 1M dataset is given in Table 3. Unlike previous random walk methodologies that randomly select the next node with equal probability, the fairness aware random walk proposed in the paper selects the next node based on the transition probability and generates a debiased metapath accordingly.

We generate meta-paths $\mathcal {P}$ of each node through the following Algorithm 1 using the pre-defined transition probability of Eq. (1).

**Algorithm 1:**
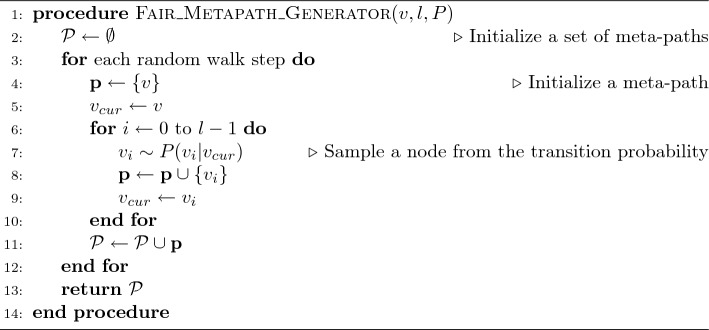
Fairness-aware Random Walker

Algorithm 1 describes the proposed meta-path generation procedure in detail. We assume that the node type of input node (i.e., first node) *v* is *User*. First, the set of meta-paths $\mathcal {P}$ is initialized (line 2). Second, a meta-path $\textbf{p}$ is initialized in each step of the random walkers (line 4). Next, the neighboring node $v_i$ of $v_{cur}$ is sampled from the transition probability *P* defined in Eq. (1) and enters set $\textbf{p}$ (line 7–8). Finally, the algorithm generates fair meta-paths $\mathcal {P}$. We generate the meta-paths *UM* and *UMUM* for each user through Algorithm 1, where *l* of *UM* is 2 and *l* of *UMUM* is 4.

We generate a dense latent vector for user *u* as follows^10^,

2 $$\begin{aligned} \textbf{e}_{u,t} = \sigma (\textsf{MEAN} (\{\textbf{W}e_j + b : j \in \mathcal {P}_{u,t}\})), \end{aligned}$$

where $\mathcal {P}_{t, u}$ is the set of meta-paths with the meta-path type *t* for user *u*, W is initialized using Xavier^51^, $\sigma$ is the activation function, and $\textsf{MEAN}(\cdot )$ is mean pooling. Afterward, we aggregate de-biased dense user representations of each user, which is formulated,

3 $$\begin{aligned} \textbf{x}_u = \sum _{t \in T} \textbf{a}_t \cdot \textbf{e}_{u,t}, \end{aligned}$$

where *T* denotes the set of meta-path types, and $\textbf{a}_t$ denotes the weight of the meta-path type *t*. We set $\textbf{a}_t$ to 1/|*T*| for all *t* in our experiments in section “Experiments”.

### Joint training with co-regularization

#### Co-regularizer

We present a novel fairness regularizer and design a joint training method^30^ to minimize the trade-off between relevance and fairness performance. The proposed fairness regularizer is formulated as the relative standard variance of the average predicted score of each group as follows,

4 $$\begin{aligned} \mathcal {L}_{F} = \frac{std \left( \mathbb {E} \left[ \hat{y}_u \right] _{g_1}, \ldots , \mathbb {E} \left[ \hat{y}_u \right] _{g_{|G|}} \right) }{mean \left( \mathbb {E} \left[ \hat{y}_u \right] _{g_1}, \ldots , \mathbb {E} \left[ \hat{y}_u \right] _{g_{|G|}} \right) }, \end{aligned}$$

where $\hat{y}_u$ denotes the set of predicted scores of items for user *u*, $g_k$ means *k*-th group and $k \in \{1, \cdots , |G|\}$. $\mathbb {E}[\hat{y}_u]_{g_k}$ is the average predicted score for items belonging to group $g_k$ among items rated by user *u*. The regularizer $\mathcal {L}_F$ encourages the recommendation model to learn that each user rates items fairly regardless of the group of items.

The loss function of relevance is mean squared error^52^, which is formulated as,

5 $$\begin{aligned} {\mathcal {L}_{R} = \frac{1}{|I_u|} \sum _{i \in I_u} \left( y_{ui} - \hat{y}_{ui} \right) ^2}, \end{aligned}$$

where $I_u$ is the set of items rated by user *u*, and $y_{ui}$ and $\hat{y}_{ui}$ denotes the actual and the predicted score rated by the user *u* to item *i*, respectively. We learn the item preference for each user by minimizing the relevance loss function $\mathcal {L}_R$. The final loss function $\mathcal {L}$ is calculated as follows,

6 $$\begin{aligned} \mathcal {L} = (1-\gamma ) \mathcal {L}_R + \gamma \mathcal {L}_F, \end{aligned}$$

where $\gamma$ is the fairness weight that controls the importance of fairness and 0 $\le \gamma \le$ 1.

#### Fairness-aware meta-learner

K-shot fairness^30^ for learning from a few data for new tasks aims to: (1) learns both the fairness and accuracy of recommendations quickly at the same time, (2) enables tuning to achieve different balances between accuracy and fairness to minimize the trade-off between performance of both. The task-specific learner learns using the support set to adapt to each task quickly, and the meta-learner absorbs knowledge about the tasks learned by the task-specific learner and updates the global parameter $\theta$. The objective of FaRM is defined as

7 $$\begin{aligned} \theta ^* = \underset{\theta }{argmin}\ \, \mathbb {E}_{\mathcal {T}_u \sim p(\mathcal {T}_u)}[(1-\gamma ) \mathcal {L}_R(f_{\theta }) + \gamma \mathcal {L}_F(f_{\theta })], \end{aligned}$$

where *f* denotes the recommendation model, and we employ a multi layer perceptron^53^ with two layers.

In detail, The local parameter $\theta _i$ will be optimized through backpropagation of the final loss function for the support set, as follow,

8 $$\begin{aligned} \min _{\theta _i} \sum _{\mathcal {T}_u \in \mathcal {T}} \mathcal {L}(\theta _i; \mathcal {S}_u). \end{aligned}$$

Similarly, the global parameter $\theta$ will be optimized through backpropagation of the query loss, as follow,

9 $$\begin{aligned} \min _{\theta } \sum _{\mathcal {T}_u \in \mathcal {T}} \mathcal {L} (\theta - \eta \nabla \mathcal {L}(\theta ; \mathcal {S}_u); \mathcal {Q}_u). \end{aligned}$$

The ultimate goal of FaRM is to quickly adapt new users and items to recommendation model *f*, minimizing degradation of relevance performance and increasing fairness performance.

## Experiments

In this section, we demonstrate that our model is superior by comparing it with other baseline models.

### Experimental setup

**Table 1:** Statistics of the Movielens dataset.

# Users	6040
# Items	3881
# Ratings	1,000,209
Sparsity	95.7331 %
User attributes	Gender, Age, Occupation, Zip code
Item attributes	Genre, Rate

#### Dataset

We experiment using Movielens 1M dataset^12^, a benchmark dataset for recommendation models. Table 1 shows statistics for the Movielens dataset. The dataset contains 6040 users, 3881 movies, and 1,000,209 rating data ranging from 1 to 5. In Table 1, the underlined attributes represent sensitive attributes for users and items. User attributes contain gender, age, occupation, and zip code, and the user-sensitive attribute, gender, is a binary group. Item attributes include genre, publishing year, age group, director, and actor, and the genre is the item-sensitive attribute.

**Table 2:** Gender-based statistics of movie genres in Movielens 1M dataset.

	Romance	Action	Sci-Fi	Musical	Crime	Adventure	Thriller
Avg (ratings_female_)	84.20	72.10	41.36	12.10	61.74	71.31	50.82
Avg (ratings_male_)	73.22	107.26	58.72	10.22	82.08	92.42	68.39
Avg (preferences_female_)	47.35	36.78	20.95	8.89	35.28	40.28	27.54
Avg (preferences_male_)	39.67	52.79	30.23	6.96	45.77	50.43	37.58

**Table 3:** Fairness-aware transition probability matrix for Movielens 1M dataset.

	Male	Female
War	0.0000	1.0000
Western	0.0000	1.0000
Sci-Fi	0.2398	0.7602
Action	0.2871	0.7129
Adventure	0.3826	0.6174
Biography	0.4000	0.6000
Horror	0.4087	0.5913
Crime	0.4256	0.5744
Thriller	0.4373	0.5627
Fantasy	0.5128	0.4872
Drama	0.5195	0.4805
Comedy	0.5458	0.4542
Film-Noir	0.6000	0.4000
Animation	0.6618	0.3382
Mystery	0.7368	0.2632
Musical	0.8333	0.1667
Romance	0.8402	0.1598
Family	0.8429	0.1571
Documentary	1.0000	0.0000

Table 2 shows gender-based statistics for the movie genre, an item-sensitive attribute. We chose six genres with gender imbalances: *Romance*, *Action*, *Sci-Fi*, *Musical*, *Crime*, *Adventure*, and *Thriller*. The female group rated *Romance* and *Musical* movies more than the male group. On the other hand, both the female and male groups rated the *Action*, *Sci-Fi*, *Crime*, *Adventure*, and *Thriller* genres a lot, but the male group rated a lot more. Each group’s preference is also similar to the average number of ratings.

Similar to existing meta-learning-based studies^9,10^, we eliminate users who rated less than 13 movies or more than 100 movies. We construct the query set $\mathcal {Q}_u$ by randomly selecting 10 items rated by each user and construct the support set $\mathcal {S}_u$ with the remaining items. We generate fair meta-paths *UM* and *UMUM* through Algorithm 1 for each task $\mathcal {T}_u=(\mathcal {S}_u, \mathcal {Q}_u)$, where $u \in U$. The fairness-aware transition probability shown in Table 3 is calculated through Eq. (1).

We construct four experimental scenarios to evaluate performance in warm-start and cold-start environments: Warm-start state (WS) with existing users and items, User Cold-start (UC) state with new users and existing items, Item Cold-start (IC) state with existing users and new items, and User-Item Cold-start (UIC) State with new users and new users. We evaluate the performance of the proposed model for four experimental scenarios in section “Performance evaluation”, and we assume the user-item cold start (UIC) environment in sections “Model analysis” and “Parameter analysis”.

#### Evaluation metrics

We adopt relevance and fairness metrics to evaluate FaRM. We use Mean Absolute Error (MAE) and Normalized Discounted Cumulative Gain at rank K (NDCG@K) as relevance metrics, and we set K=5. We use Accuracy and Macro F-Score as fairness metrics^11^, where smaller values denote better fairness performance with less impact on sensitive attributes. The lower the value of these classification metrics, the less influence of sensitive attributes in the learning process.

#### Compared methods

We compare FaRM with three existing methods: MetaHIN^10^, Random and NFCF^15^. MetaHIN is a model that improves the accuracy of recommendations by introducing a heterogeneous information network to a meta-learning-based cold-start recommendation model. Random and NFCF use it as baseline models to evaluate the fairness of FaRM. Random is suitable as a baseline model for comparing fairness performance because it randomly estimates user preferences regardless of sensitive attributes. NFCF is a fairness-aware recommendation model that enhances fairness to Neural Collaborative Filtering (NCF)^21^.

#### Parameter settings

We adopt Adaptive Moment Estimation (Adam) for optimization, and we set the batch size to 32 and the maximum number of epochs to 100. The model *f* consists of two fully-connected layers, and we set the hidden dimension of each layer to 64. We construct the embedding vectors for each attribute of the user and item and set the dimension of all embedding vectors to 32. We set both learning rates for local update and global update to 0.001, and set the fairness weight $\gamma$ in Eq. (6) to 0.5. We experiment with the impact of the hyperparameter $\gamma$ on the performance of FaRM in section “Parameter analysis”.

### Performance evaluation

We compare FaRM and different comparative models in four experimental scenarios (i.e., WS, UC, IC and UIC) in this section. Table 4 shows the results of the performance comparison experiments on the Movielens 1M dataset.

**Table 4:** Experimental results of relevance and fairness performance for different models in 4 scenarios.

Scenario	Model	MAE	NDCG@5	Macro-F	Accuracy
Warm Start (WS)	MetaHIN	0.9003	**0.8735**	0.5033	0.5864
	Random	1.5465	0.6751	0.4395	*0.4800*
	NFCF	**0.7273**	*0.8148*	*0.3801*	**0.4623**
	FaRM	*0.8561*	0.8086	**0.3668**	0.4908
User Cold-start (UC)	MetaHIN	*0.8947*	**0.8668**	0.4946	0.5771
	Random	1.4083	0.6718	0.4130	*0.4531*
	NFCF	0.9076	0.7073	*0.4020*	0.4679
	FaRM	**0.8776**	*0.7962*	**0.3663**	**0.4474**
Item Cold-start (IC)	MetaHIN	*0.9966*	**0.8038**	0.4900	0.5559
	Random	1.5259	0.6851	*0.4385*	0.4774
	NFCF	1.7800	0.6309	0.3590	*0.4687*
	FaRM	**0.9297**	*0.7784*	**0.3396**	**0.4608**
User-Item Cold-start (UIC)	MetaHIN	*1.0009*	**0.7934**	0.4829	0.5514
	Random	1.6207	0.6368	0.4453	0.4864
	NFCF	2.4544	0.6319	*0.3354*	*0.4569*
	FaRM	**0.9998**	*0.7658*	**0.3267**	**0.4565**

The best model is bolded, and the second-best model is the italic.

#### Fairness performance

Our method achieves the best performance for all fairness metrics in three cold start scenarios (i.e., UC, IC, and UIC). In detail, FaRM outperforms NFCF by 3.6%, 5% and Random by 2%, 0.5% on Macro-F and Accuracy in UC scenarios with new users and existing items. FaRM significantly improves fairness performance compared to other methods in both IC and UIC scenarios. These results show that FaRM contributes significantly to improving fairness in the cold-start states. On the other hand, in the warm start scenario (i.e., WS), NFCF outperforms FaRM on Accuracy, but FaRM shows the best fairness performance on Macro-F. In particular, the experimental results show that FaRM performs much better than Random in Macro-F. Even though Random is a strong baseline, FaRM has a higher fairness performance than Random in most scenarios because the data distribution is unfair, as shown in Table 2. Random determines the fairness of the recommendation result according to whether the training data distribution is fair or unfair. In contrast, our model achieves higher fairness performance than Random by corresponding to the distribution by genre regardless of raw data distribution. These results imply that FaRM can generally improve fairness in all scenarios.

#### Relevance performance

MetaHIN showed the best performance in all states for the relevance metric, NDCG@5, while the proposed method in all cold states outperformed MetaHIN on MAE. Furthermore, our method showed significantly higher performance on NDCG@5 than two fairness-aware models in all four scenarios and the best performance on MAE in all cold states. NFCF achieves the best performance on MAE in the warm-start state (WS), while it achieves similar to or lower relevance performance than Random in three cold-start environments (i.e., UC, IC, and UIC). These results show that NFCF performs poorly in cold-start scenarios because it is a warm-start model that does not consider new users or items. In contrast, the proposed method achieves the highest performance on MAE in three cold-start environments (i.e., UC, IC, and UIC). Our fairness-aware model recommends the most widespread war movies, even those who do not like war movies. Therefore, FaRM eliminates bias while improving the relevance performance and increases MAE performance by minimizing overfitting. This experiment shows that FaRM is suitable for reducing loss of relevance performance while increasing fairness performance in cold start states. Thus, FaRM significantly improves fairness performance by minimizing the trade-off between relevance and fairness.

### Model analysis

**Figure 6:**
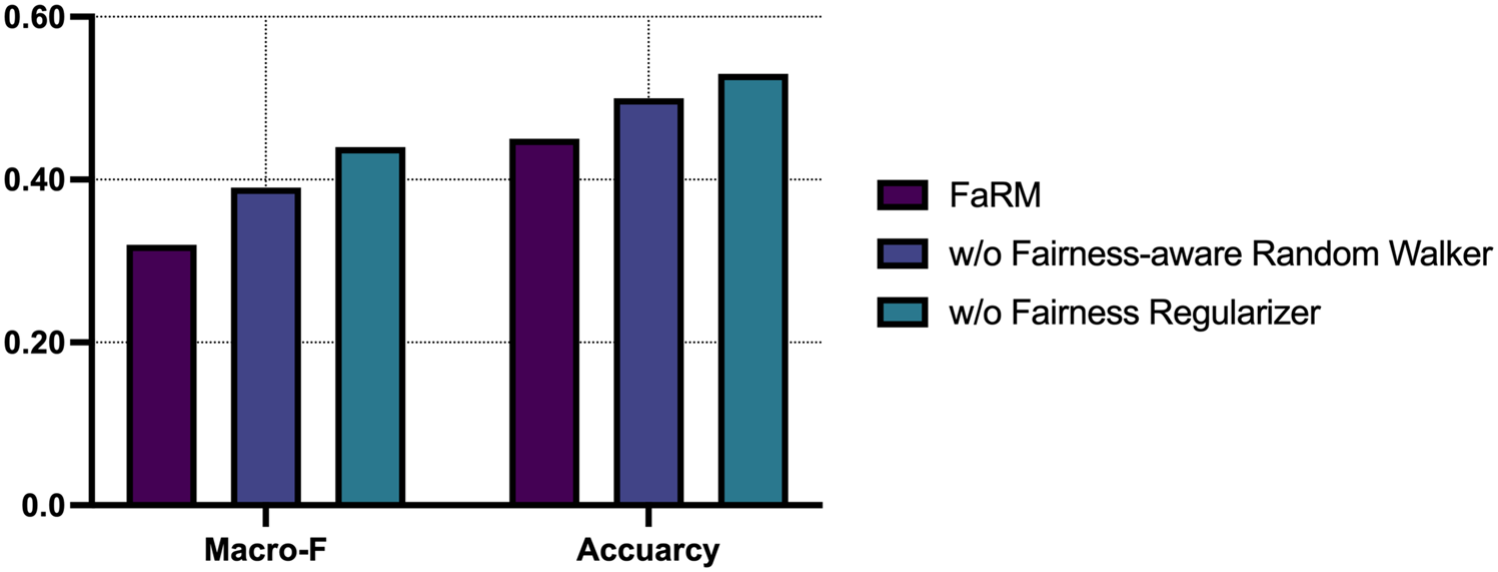
The effectiveness of each component of FaRM. Lower scores indicate better fairness.

We analyze the fairness performance of each component of FaRM in the user-item cold start environment (UIC). In Fig. 6, it is shown fairness performance on Macro-F and Accuracy without each component of FaRM. The fairness performance of FaRM (i.e., including all components) is the highest, which means that all components of FaRM are essential. In other words, we demonstrate that all components of the proposed model play an important role in improving fairness performance. We also find that the fairness regularizer is crucial for improving fairness. This shows that the recommendation model learns fairness appropriately through the fairness regularizer. We also find that the impact on the fairness-aware random walker is quite significant. This is because the fairly generated meta-path can reduce bias for the sensitive attribute of the user.

### Parameter analysis

**Figure 7:**
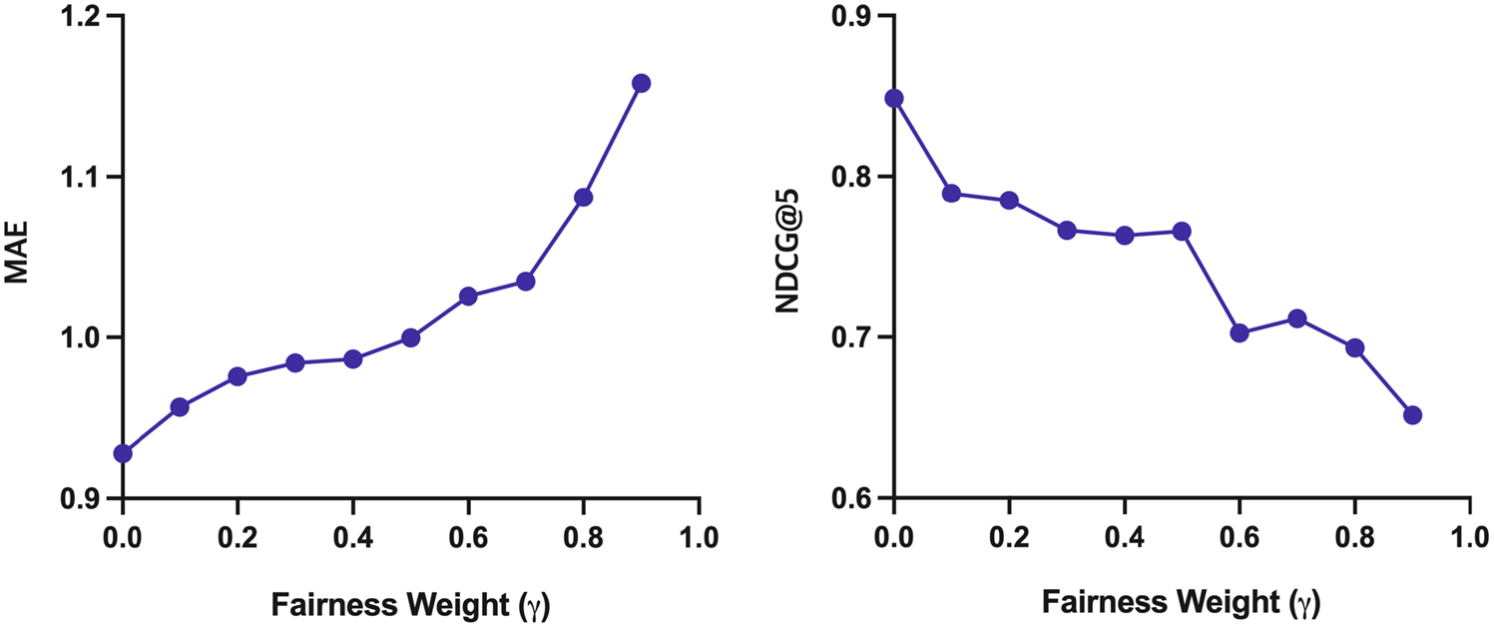
The relevance performance w.r.t. different $\gamma$.

**Figure 8:**
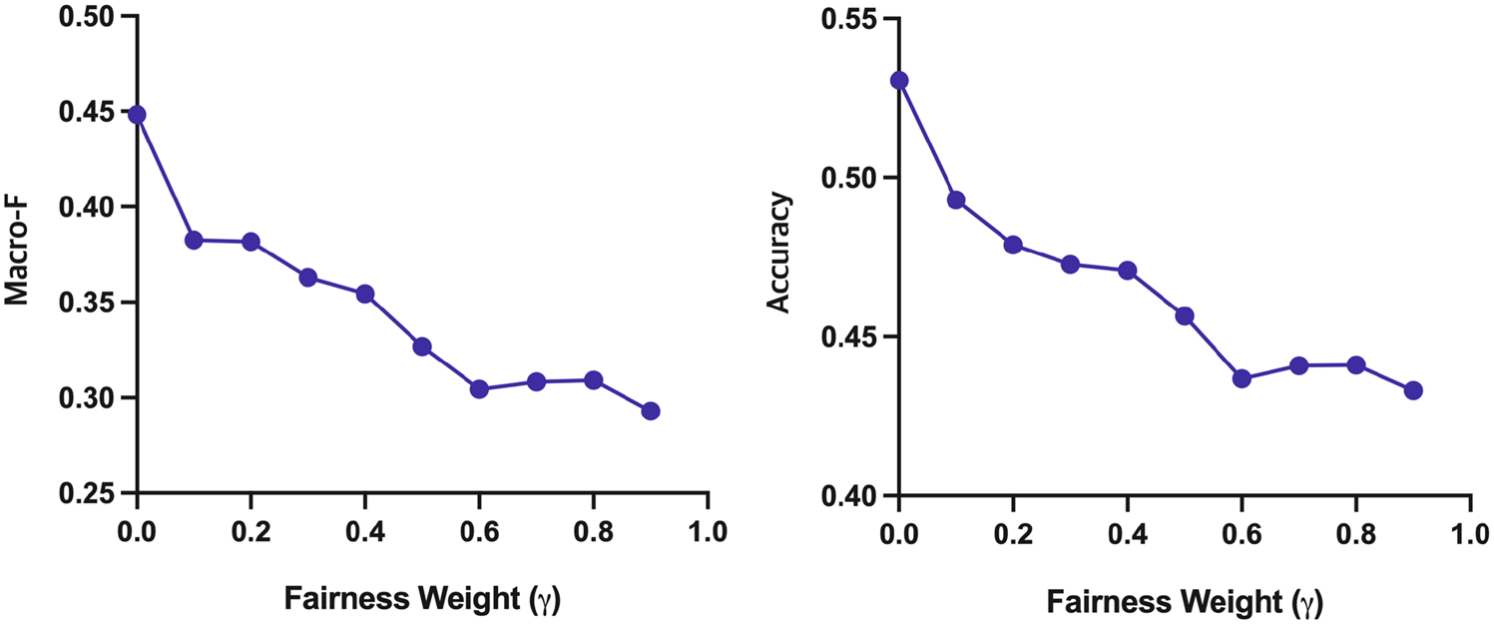
The fairness performance w.r.t. different $\gamma$.

Figures 7 and 8 show the relevance and fairness performance according to fairness weight $\gamma$ in Eq. (6), respectively. The x-axis of each graph represents the hyperparameter $\gamma$ and ranges from 0 to 1. In Fig. 7, it is shown that the relevance performance of FaRM decreases as the fairness weight increases. On the other hand, the performance of the Macro-F and Accuracy increase as the fairness weight increases, as shown in Fig. 8. These results show the influence of fairness weights $\gamma$ on fairness performance. We find that the NDCG@5 significantly declines when the fairness weight is more than 0.6. We also find that the fairness performance does not improve significantly when the fairness weight is 0.6 or higher. Therefore, we set $\gamma$ to 0.5 to minimize the trade-off between relevance and fairness performance.

## Conclusion

In this paper, we propose a novel meta-learning-based recommendation framework to improve the fairness of recommendation models in cold-start environments. We propose a novel fair meta-paths generation algorithm and fairness regularizer and introduce joint training on relevance and fairness objectives. In addition, each component of the proposed framework can be used by all models that require improving group fairness. Extensive experiments demonstrate that the proposed model outperforms state-of-the-art cold-start and fairness-aware recommendation models for relevance and fairness in various cold-start scenarios.

## Data Availability

Harper, F., Konstan, J.: The movielens datasets: History and context. ACM Transactions on Interactive Intelligent Systems 5(4) (2015) https://doi.org/10.1145/2827872^12^.
